# Effects of Temperature on the Characteristics of Nitrogen Removal and Microbial Community in Post Solid-Phase Denitrification Biofilter Process

**DOI:** 10.3390/ijerph16224466

**Published:** 2019-11-13

**Authors:** Qian Zhang, Xue Chen, Wandong Luo, Heng Wu, Xiangyang Liu, Wang Chen, Jianhong Tang, Lijie Zhang

**Affiliations:** 1 School of Chemistry and Chemical Engineering, Chongqing University of Technology, Chongqing 400054, China; zhangqianswu2005@163.com (Q.Z.); 15823108783@163.com (X.C.); lwd265588@163.com (W.L.); wuhengdyx@163.com (H.W.); liuxy@2017.cqut.edu.cn (X.L.); 18323050874@163.com (W.C.); nidhogg_t@163.com (J.T.); 2 School of Pharmacy and Bioengineering, Chongqing University of Technology, Chongqing 400054, China

**Keywords:** biodegradable polymers, gas/water ratio, microbial community, PCR-DGGE, post solid-phase denitrification

## Abstract

In order to solve the problems of high energy consumption, complex process and low nitrogen removal efficiency in the currently available low carbon source wastewater treatment processes, a novel coagulation sedimentation/post-solid-phase denitrification biofilter process (CS-BAF-SPDB) was proposed. The effect of temperature on the nitrogen removal performance of BAF-SPDB was intensively studied, and the mechanism of the effect of temperature on nitrogen removal performance was analyzed from the perspective of microbial community structure by using the polymerase chain reaction denaturing gradient gel electrophoresis (PCR-DGGE). The results showed that, to realize favorable nitrifying and denitrifying performance simultaneously in the BAF-SPDB unit, the operation temperature should be set above 18 °C. In addition, the influence of the macro operation parameters on the performance of the BAF and SPDB has a direct relationship with the dynamic changes of the micro microbial community. The influence of temperature on nitrification performance in BAF was mainly embodied in the change of composition, amount and activity of ammonia oxidizing bacteria *Candidatus Nitrospira defluvii* and nitrite oxidizing bacteria *Nitrosomonas sp. Nm47*, while that on denitrification performance in SPDB is mainly embodied in the change of composition and amount of solid carbon substrate degrading denitrifying bacteria *Pseudomonas* sp., *Myxobacterium AT3-03* and heterotrophic denitrifying bacteria *Dechloromonas agitate*, *Thauera aminoaromatica*, *Comamonas granuli* and *Rubrivivax gelatinosus*.

## 1. Introduction

In recent years, municipal domestic wastewater has become characterized by a low carbon to nitrogen (C/N) ratio [1]. Several treatment ways of nitrogen have been established, such as physical/chemical method, which normally require high operational cost, low selectivity, and produce brine wastes [2]. Sulfur-based autotrophic denitrification processes were also a method of nitrogen removal, but as Hao et al. and Qiu et al. reported, it has a complicated sulfur oxidation process which is difficult to control [3,4], presenting serious problems for nitrogen removal from wastewater, e.g., high operation costs caused by excessive external carbon source dosage and high recycle ratio, sludge bulking under low organic loading and low dissolved oxygen and poor nitrogen removal efficiency [5,6,7].

In this study, we propose a novel post denitrification strategy based on solid-phase denitrification to realize high nitrogen removal efficiency from wastewater with a low C/N ratio. This strategy is termed coagulation sedimentation-post solid-phase denitrification biofilter process (CS-BAF-SPDB). In the first step, most of the suspended solids and organic matter are removed in the coagulation sedimentation unit (CS), which could not only ease the blockage of the subsequent biological filters unit, but also to avoid the impact of organic matter on the nitrification. In the second step, ammonia nitrogen is oxidized to nitrate nitrogen in the biological aeration filter (BAF). Compared with the activated sludge process, the advantage of BAF is that the biomass of nitrifying microorganism is guaranteed, and sludge bulking would not occur even under low organic carbon loading and low dissolved oxygen. The effluent of BAF then flows into the solid-phase denitrification biofilter (SPDB), and the nitrate nitrogen in the effluent is converted to N_2_. Considering the biodegradability, the start-up cycle and the stability of the degradation process, we have chosen the biodegradable polymer polycaprolactone (PCL) instead of other biodegradable polymers in SPDB [8,9,10,11,12], as PCL is used as both a biofilm support and a carbon source. Denitrifying bacteria in the biofilm coating onto the surface of the PCL carrier could degrade PCL to acquire the necessary electron donor for denitrification. In addition, the organic carbon released from PCL could also eliminate the dissolved oxygen in the influent to maintain the anoxic environment. Previous studies have also shown that the solid carbon source has a high mechanical strength and a very long service life (mass loss of 0.03% per day), so that a long-term stable denitrification performance can be maintained in SPDB [13]. In previous research, using various biodegradable polymers (e.g., PHB, PCL, PLA and polymer blends) as both a carbon source and biofilm support for treating nitrate contaminated water in a single reactor (e.g., packed-bed and moving bed biofilm reactor, constructed wetland, etc.) has been widely studied [8,9,10,11,12]. However, research on using the combination process of SPDB and BAF for low carbon domestic wastewater treatment has rarely been reported.

Temperature is one of the key factors affecting nitrogen removal efficiency of BAF-SPDB process [14,15]. It not only affects the activity, specific growth rate and community structure of nitrifying and denitrifying microorganisms, but also has a certain impact on the concentration of dissolved oxygen, and thereby has an impact on nitrification and denitrification. Therefore, to determine the appropriate operating temperature is the key to ensure the ideal nitrogen removal efficiency of this process. However, the effect of temperature on the combination process of SPDB and BAF for low carbon wastewater treatment has rarely been reported. In addition, little information is available on the functional bacteria predominating in this nitrogen removal process and the change of microbial community structure in BAF and SPDB with changing temperature, so further research is necessary. 

The start-up of this novel process and the effect of influent carbon/nitrogen ratio, gas/water ratio, hydraulic detention time and influent ammonia loading on the nitrogen removal efficiency of BAF-SPDB was investigated in the preliminary study in our laboratory [16]. On this basis, this paper focuses on the influence of temperature on process’ nitrogen removal efficiency, and the changes of microbial community structure with temperature are studied by using a polymerase chain reaction-denaturing gradient gel electrophoresis (PCR-DGGE) technique to provide the microbiological basis for the research of process optimization and nitrogen removal mechanism.

## 2. Materials and Methods

### 2.1. Materials

Polycaprolactone (PCL) and clay ceramsite carriers were kindly supplied by Shenzhen Esun Industrial Co. Ltd and Jiangxi Pingxiang Sanhe Ceramics Co., Ltd. Their physical–chemical properties are shown in Table 1.

Domestic wastewater from student dormitories of Chongqing University, China (29.611011N, 106.299419E) was used as feed water. The water quality parameters of the influent were as follows: ρ(COD_Cr_) = 148–185 mg/L, ρ(NH_4_^+^-N) = 26.7–56.8 mg/L, ρ(TN) = 28.6–70.2 mg/L, ρ(SS) = 140.0–152.5 mg/L. To investigate the effect of operation conditions on nitrogen removal efficiency of the process, COD_Cr_, NH_4_^+^-N and PO_4_^−^-P concentration in the feed water were controlled by dilution or adding sodium acetate ammonium chloride and potassium dihydrogen phosphate to maintain the stability of water quality.

Activated sludge, obtained from the aerobic and anaerobic tank of A^2^/O process in Yongchuan Sewage Plant, Chongqing, China (29.397933N, 105.965335E), was used as seed source for BAF and SPDB, and the concentration of the activated sludge was 4.2 g/L and 5.6 g/L, respectively.

### 2.2. Experimental Apparatus 

**Figure 1:**
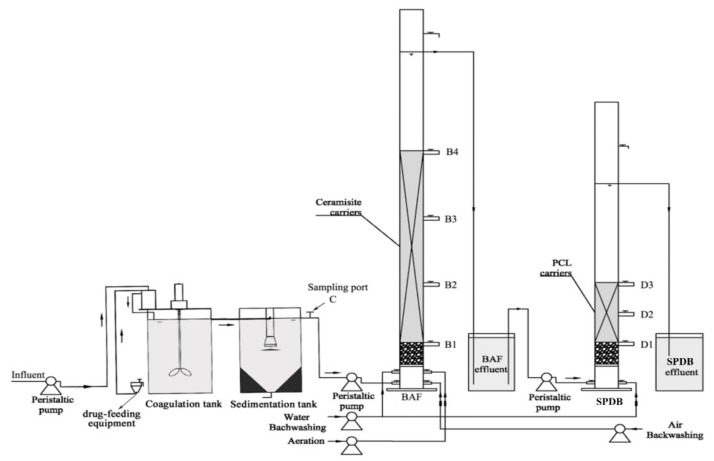
Schematic diagram of the sedimentation/post-solid-phase denitrification biofilter (CS-P-SPDB) process cascade.

Figure 1 illustrates the schematic diagram of the CS-BAF-SPDB process, which combined the coagulation sedimentation unit with the biofilter system. The volume of both the coagulation and sedimentation tank was 5 L, and a sampling point (C) was set up in the effluent of the sedimentation tank. The biofilter system consisted of two cylindrical columns. The first column, which was aerated for nitrification, was 175 cm in height and had an inner diameter of 8 cm. This column was packed with ceramic balls of 0.4–0.6 cm in diameter, and the packing height was 87 cm. Four sampling points (B1, B2, B3 and B4) were set up from the bottom to the top along the BAF. The second column was in 130 cm height and has an inner diameter of 8 cm. This column is packed with PCL particles as biofilm support and electron donor, and the packing height was 27 cm. Three sampling points (D1, D2 and D3) were set up along the SPBD. The effective volume (liquid volume) of each column was 4.0 and 3.2 L, respectively. The flow rate of the influent was controlled by peristaltic pumps (Longer BT100-2J, China). The aeration rate of the BAF was controlled by gas flow meter (Senlod LZB-3, China). The water temperature was strictly controlled by electrical heating tape wrapped around the biofilter reactors.

### 2.3. Experimental Procedure and Sampling Methods 

The start-up of the CS-BAF-SPDB process was successfully achieved in the previous study [16], and the optimum operating condition, such as influent C/N ratio and gas/water ratio of BAF, the hydraulic retention time (HRT) of BAF and SPDB, were also determined. In order to study the influence of changing temperature on the nitrogen removal performance of CS-BAF-SPDB process under low carbon conditions, influent ammonia concentration was kept at 30 mg/L, C/N ratio in the effluent of CS units was maintained at 3:1, gas/water ratio of BAF was set at 4:1, the HRT of BAF and SPDB was control at 4 h and 2 h, respectively, and the temperature was changed between 32 and 13 °C. Sampling at a fixed time each day, once the effluent ammonia, nitrate and TN concentration from three continuous daily tests were within 5% of each other, the reactor was assumed to be operated at steady state condition under a certain temperature and the temperature was then changed into another level. In a previous study, it was confirmed that the nitrifying bacteria predominated in the B3 sampling point of BAF, and denitrifying bacteria mainly predominated in D2 sampling point of SPDB [16]. Therefore, biofilm samples from B3 and D2 were more representative. When the process operated at steady state condition under each temperature, ceramsite and PCL carriers with biofilm coating onto the surface were taken from B3 and D2 sampling points, respectively. Biofilms were removed by ultrasonic device, and the samples were preserved at −20 °C for further analysis. The morphology of the solid samples was observed using an environmental scanning electron microscope (ESEM) (Quanta 200 FEG, Hillsboro, OR, USA).

### 2.4. Extraction of Sample Genomic DNA

Genomic DNA samples were extracted by using rapid extraction kit of silt genomic DNA (Baisaike, Cat#DP4011, Beijing, China).

### 2.5. PCR Amplification of 16S rDNA Fragments in Bacterial

Taking sample genomic DNA as templates, hypervariable region sequences of samples of 16S rDNA were amplified using two universal primers: GC-338F and 518R bacterial. 

System for PCR amplification (50 μL): 10 × PCR buffer 5 μL, dNTP (2.5 mM) 3.2 μL, rTaq (5 U/μL) 0.4 μL, GC-338F20 μM1 μL, 518R (20 μM) 1 μL, template DNA 50 ng, adding ddH2O to 50 μL.

Program PCR amplification: pre-denaturation at 94 °C for 5 min, denaturation at 94 °C for 1 min, annealing at 55 °C for 45 s, extension at 72 °C for 1 min, 30 cycles, and final extension at 72 °C for 10 min.

PCR products were purified and recycled using DNA Gel Extraction Kit of OMEGA.

PCR Instrument was T-gradient produced by Biometra, Gel Imager was Gel-Doc2000 produced by Bio-Rad. The universal primer information is shown in Table 2.

### 2.6. Analysis of the PCR Products by Denaturing Gradient Gel Electrophoresis (DGGE)

10 μL PCR products were taken for DGGE analysis. Electrophoresis was proceeded at 150 V, 60 °C, in 1 × TAE buffer for 5 hours with a denatured gradient from 35% to 55%, and 8% polyacrylamide gel. The chemical denaturing agent was 100% urea 7 mol/ L, along with 40% acrylamide(v/v). The level of DGGE gel formulations (Table 3) were among 35%–55% of denatured gradient.

After denaturing gradient gel electrophoresis (DGGE), the steps of silver staining were interpreted as follows:

1. Fixed for 15 min with fixative (a constant volume of 500 mL with 50 mLC_2_H_5_OH and 2.5 mL CH_3_COOH).
2. Cleaned with Milli-Q pure water, 20 s and 2 min each time.
3. Stained for 15 min with silver dye (a constant volume of 500 mL with 1 g AgNO_3_ and 0.75 mL 37% HCHO).
4. Cleaned with Milli-Q pure water for 20 s and 2 min each time.
5. Colored for 5–7 min with color solution (a constant volume of 500 mL with 7.5 g NaOH and 2.5 mL 37% HCHO).
6. Finally, the reaction was terminated with stop solution (a constant volume of 500 mL with 50 mL C_2_H_5_OH and 2.5 mL CH_3_COOH).

### 2.7. Recycling and Sequencing of the Dominant Bands in the DGGE Profiles

DGGE strips were cut and recovered with sterilized scalpel, and recycled target band using Poly-Gel DNA Extraction Kit of OMEGA. 2 μL recovered products were used as template, using 338 F/518 R as primers to PCR reactions.

PCR amplification system (50 μL):10 × PCR buffer 5 μL, dNTP (2.5 mM) 3.2 μL, rTaq (5 U/μL) 0.4 μL, 338F (20 mM) 1 μL, 518R (20 mM) 1 μL, template DNA 1 μL, adding ddH_2_O to 50 μL.

PCR amplification program: pre-denaturation at 94 °C for 4 min, denaturation at 94 °C for 30 s, annealing at 55 °C for 30 s, extension at 72 °C for 30 s, 30 cycles, and final extension at 72 °C for 10 min.

The re-amplified DNA fragments were recovered, purified and connected to the carrierpMD18-vectors. Then they were transformed into DH5α competent cells, and positive clones were screened. Finally, bacterial 16S rDNA fragments inserted in broth were sequenced by BGI.

### 2.8. Data Analysis 

The dominant DGGE bands in the lane of BAF and SPDB, which changed obviously during the temperature changing process, were successfully re-amplified, cloned, sequenced and aligned with BLAST to get access to the 16S rDNA sequence of the most similar to type strain with BLAST program for homology in GenBank. Phylogenetic tree was constructed with the neighbor-joining method by employing MEGA5 software, bootstrap index was 1000.

## 3. Results and Discussion 

### 3.1. The Influence of Temperature on Nitrogen Removal Efficiency of CS-BAF-SPDB

**Figure 2:**
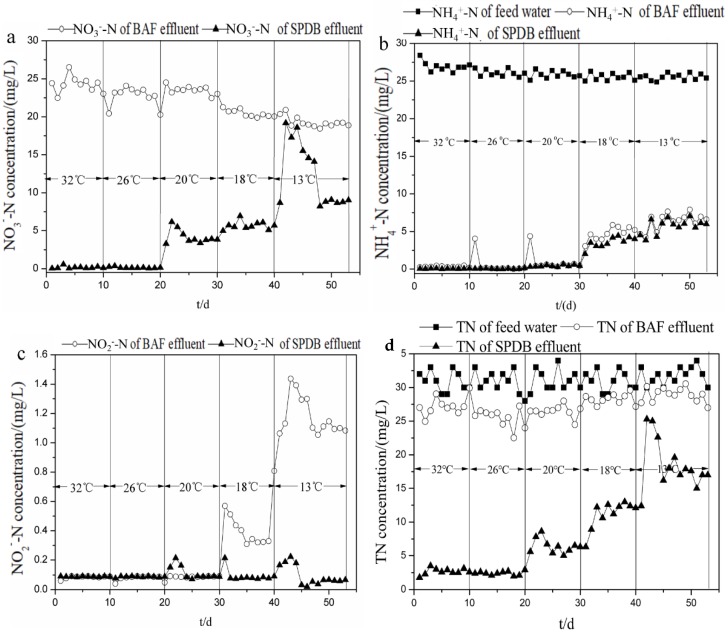
Influence of temperature on effluent (**a**) nitrate, (**b**) ammonia, (**c**) nitrite and (**d**) TN concentration of biological aeration filters (BAF) and solid-phase denitrification biofilters (SPDB).

As can be seen from Figure 2a, when the temperature is above 18 °C, changing temperature had little effect on nitrification activities, and NO_3_^−^-N in the effluent of BAF showed almost no change. However, nitrification in BAF was obviously inhibited with the temperature decreasing from 18 °C to 13 °C, and NH_4_^+^-N removal efficiency decreased from 98.0% at 18 °C to 78.1% at 13 °C (Figure 2b). This was consistent with the previous studies that NH_4_^+^-N removal efficiency decreased from 85.7–96.3% at a water temperature of 20–25 °C to 71.9–87.8% at 7–10 °C in a BAF packed with zeolite as biofilm support [17]. Antoniou et al. [18] pointed out that the effective maximum specific growth rate of nitrifying bacteria was found to be a monotonically increasing function of temperature in the range of 15–25 °C. Therefore, the decrease of temperature might affect the maximum specific growth rate of the nitrifying bacteria, and further influence the nitrification process. Similarly, when the temperature was above 26 °C, temperature changing has little effect on denitrification in the SPDB. When the temperature further decreased, however, denitrification in SPDB was obviously inhibited, and NO_3_^-^-N in the effluent of SPDB gradually increased from 0.1 mg/L at 26 °C to 8.8 mg/L at 13 °C (Figure 2a). Wang and Wang [19] also found that the nitrate removal efficiency decreased from nearly 100% at 25 °C to 50% at 12 °C with a HRT of 2 h when using biodegradable snack ware (BSM) as carbon source for denitrification. Rusmana et al. [20] found that the maximum specific growth rate of denitrifying bacteria increased with the increase of temperature, and reached its maximum when the temperature was 26 °C, which explained the little effect of changing temperature on denitrification performance when the temperature increase from 26 °C to 32 °C. In addition, the decrease in temperature leads to the increase of the DO concentration in the effluent of nitrifying biofilter, which might be another reason for the worse denitrification performance under low temperature. Therefore, the temperature of the novel process should be kept above 18 °C to obtain high nitrification and denitrification performance simultaneously. From Figure 2c, it can also be seen that when the temperature decreased from 20 to 13 °C, nitrite accumulation was observed obviously in the effluent of BAF, but the nitrite concentration in the effluent of SPDB was maintained at a very low level during the whole process. From Figure 2d, it can also be seen that when the temperature decreased from 32 to 13 °C, TN removal efficiency was 15.6% which was relatively stable in the effluent of BAF. However, the TN concentration in the effluent of SPDB was maintained at a very low level at 32 °C and 26 °C, and TN removal efficiency was 87.9%. When temperature decreased from 20 to 13 °C, TN accumulation was observed obviously in the effluent of SPDB.

### 3.2. The Analysis of DGGE Profiles 

**Figure 3:**
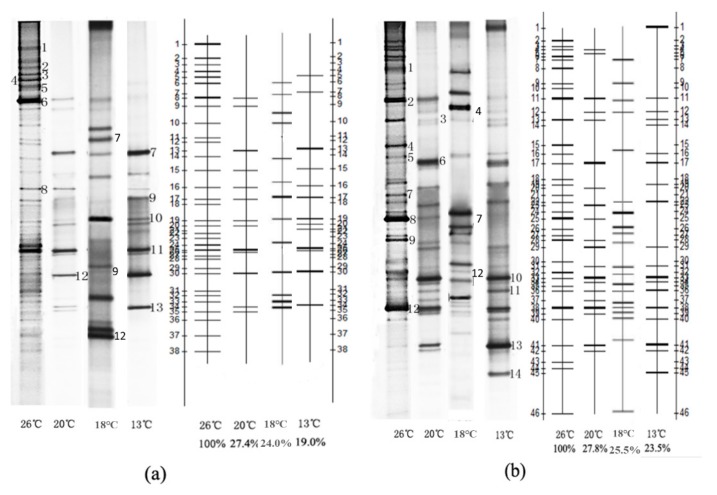
Comparison of the DGGE patterns and quantitative analysis diagrams of biofilm samples taken from different gas/water ratio conditions: (**a**) BAF; (**b**) SPDB.

Figure 3a shows the dynamic change process of microbial communities in the BAF at temperatures of 26, 20, 18 and 13 °C, respectively. The band numbers of each lane at the temperature 26, 20, 18, 13 were 33, 13, 13 and 13, respectively. The diversity of microbial communities in BAF decreased dramatically when the temperature decreased from 26 to 20 °C and then remained stable as the temperature further decreased to 13 °C, indicating that the bacteria abundance in BAF fell sharply when the temperature was less than 20 °C. Similar results were also obtained in the previous researches by Zhu et al. [21].

As can be seen from quantitative analysis diagrams in Figure 3a, DGGE bands 8, 9, 10, 11 and 12 existed in all the four lanes, indicating that the strains corresponding to these bands have strong adaptability and wide ecological amplitude, and thereby can survive under different temperature conditions. The band signal of band 8 decreased gradually, and band 11 changed little, while bands 9, 10 and 12 increased gradually, suggesting that temperature had a certain influence on corresponding strains. DGGE bands 7 and 13 only appear in the last three lanes, and with the decrease of temperature the corresponding strains gradually become the dominant bacteria, which means that these microorganisms have a strong ability to resist low temperature, and have more competitive advantages at low temperature. DGGE bands 1, 2, 3 and 5 only appeared in lane 1, indicating that these strains had harsher environment demand, and were eliminated at low temperature due to the loss of competitive advantage. DGGE band 6 on behalf of strains was the dominant bacteria in lane 1, but with decreasing temperature this strain gradually disappeared from the lane too. 

Figure 3b shows the dynamic change process of microbial communities in the SPDB at temperatures of 26, 20, 18 and 13 °C, respectively. The band numbers of each lane at the temperature 26, 20, 18, 13 were 33, 20, 20 and 19, respectively. In SPDB, the variation trend of microbial diversity with temperature was in consistent with that of BAF, but the decreasing range of microbial diversity in SPDB is lower than that of BAF. 

As was shown in the quantitative analysis diagrams of Figure 3b, DGGE bands 2, 3, 6, 10, 11, 12 and 13 existed in all the three lanes, indicating that the strains corresponding to these bands have strong adaptability and wide ecological amplitude, and can survive under different temperature conditions. The band signal of bands 2 and 3 decreased gradually, bands 11 and 13 increased gradually, and band 12 changed little, while bands 6 and 12 increased firstly and then kept invariant. Therefore, it can be inferred that temperature had a certain influence on corresponding strains. DGGE bands 1, 4, and 7 only appeared in lane 1, indicating that these strains had harsher environment demand, and were eliminated at low temperature due to the loss of competitive advantage. The band signals of bands 8 and 9 decreased firstly and then disappeared as temperatures decreased, indicating that the strains represented by these bands can survive only within a certain temperature range, and cannot survive at low temperatures. DGGE band 14 with intensive signals only existed in the third lane, suggesting that the corresponding strains were cold resistant bacteria that can only survive at low temperature. 

### 3.3. Sequencing and Discussion 

**Figure 4:**
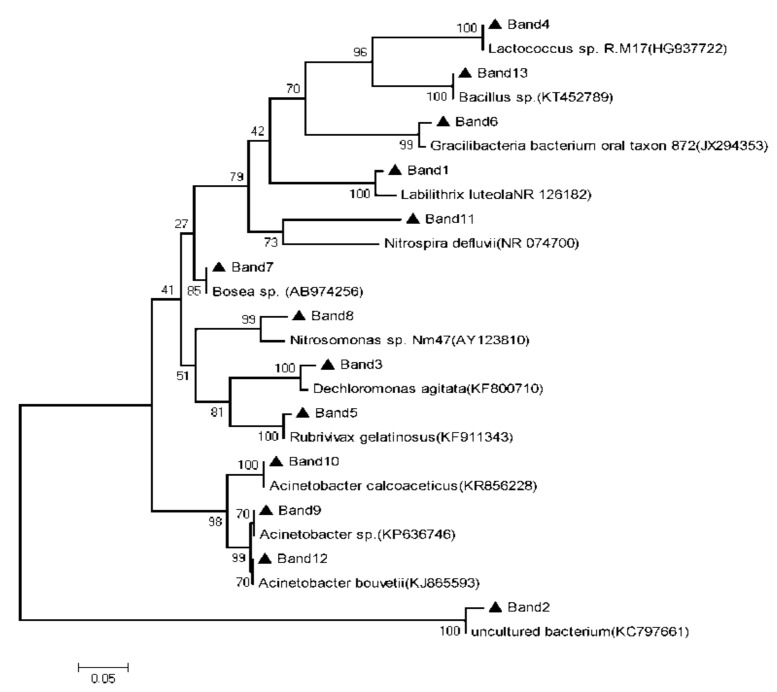
The phylogenetic tree of the main colonial species in BAF.

**Figure 5:**
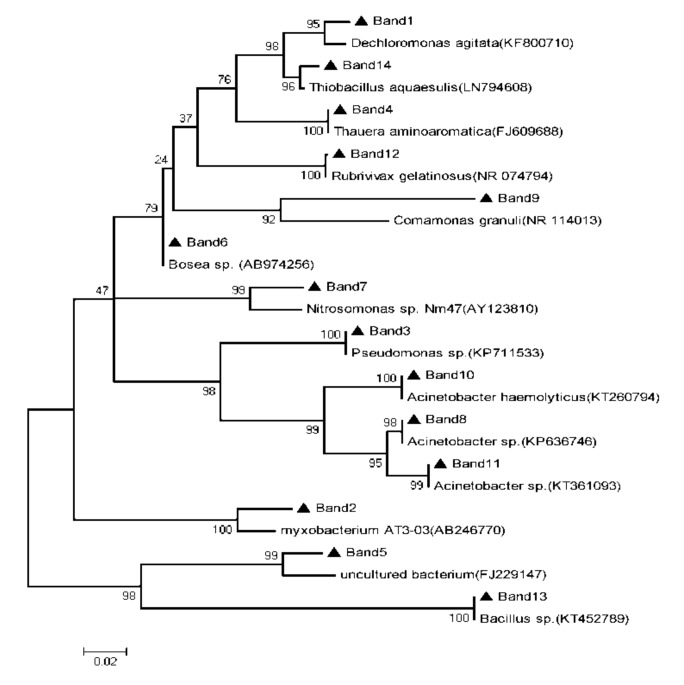
The phylogenetic tree of the main colonial species in SPDB.

Table 4 and Table 5, Figure 4 and Figure 5 shows the analysis results of DGGE gel bands recovery sequence and the phylogenetic tree of the main colonial species in BAF and SPDB, respectively. 

As can be seen from Table 4 and Figure 4, DGGE bands 8 and 11 showed 95% and 99% similarity to *Nitrosomonas sp. Nm47* and *Candidatus Nitrospira defluvii*, respectively, which are important ammonia oxidizing bacteria and nitrite oxidizing bacteria found in wastewater biological treatment system [22,23]. When the temperature decreased from 26 to 20 °C, the band signals of bands 8 and 11 changed little, indicating that the reduction of temperature in a certain range would not significantly affect the composition and biomass of nitrifying bacteria. When the temperature further decreased from 20 to 13 °C, however, the brightness of strip 8 declined obviously, while that of strip 11 changed slightly, suggesting that the growth of ammonia oxidizing bacteria was restrained under low temperature conditions, and the biomass of microorganisms therefore decreased, but the growth rate of nitrite oxidizing bacteria in the temperature range of 5–20 °C was much higher than that of ammonia oxidizing bacteria, so the effect of low temperature on the growth of nitrite oxidizing bacteria was much less than that of ammonia oxidizing bacteria. As can be seen from the nitrification performance of BAF under different temperatures in Figure 3, the ammonia nitrogen and nitrite nitrogen concentration in the effluent of BAF maintained at a low level, while the effluent nitrate concentration remained at a high level when the temperature decreased from 26 to 20 °C. The stability of the biomass and microbial composition of nitrifying bacteria mentioned above might be the main reason for the stable nitrification performance of BAF during the temperature decreasing process. It can also be inferred that the nitrification activity of nitrifying bacteria was not influenced when the temperature decreased from 26 to 20 °C. When the temperature continued to decrease from 20 to 13 °C, the concentration of ammonia nitrogen and nitrite nitrogen in the effluent of BAF increased obviously. Due to the decrease in the biomass of ammonia oxidizing bacteria, the oxidation process of ammonia nitrogen is inhibited to some extent, and therefore ammonia nitrogen in the influent could not be completely oxidized to nitrite. Although the biomass of nitrite oxidizing bacteria changed little, the nitrification activity is affected by lower temperature. Therefore, nitrite produced by ammonia oxidizing bacteria could not be further oxidized to nitrate in time, resulting in the accumulation of nitrite to some extent. DGGE bands 9, 10 and 13 displayed 100% similarity with *Acinetobacter* sp., *Acinetobacter calcoaceticus* and *Bacillus* sp., respectively, all of which are heterotrophic nitrifying bacteria. *Acinetobacter* sp. comes mainly from the ABS wastewater treatment system, which can oxidize ammonia nitrogen to nitrite and nitrate [24]. *Bacillus sp*. is a heterotrophic nitrification-aerobic denitrifying bacteria isolated from MBR system. It can not only oxidize ammonia nitrogen to nitrite, but also reduce nitrite to nitrogen [25]. *Acinetobacter calcoaceticus* can convert ammonia to nitrate, but could not reduce nitrate to nitrite or nitrogen gas under aerobic conditions [26]. The biomass of all these microorganisms increased gradually with the decrease of temperature, and gradually developed into a dominant flora, indicating that these microorganisms have a strong ability to resist low temperature, and have more competitive advantages at low temperature. Previous research proved that *Acinetobacter* sp., *Acinetobacter calcoaceticus* and *Bacillus* sp., all showed capacity of heterotrophic nitrification under very low temperature due to the regulation of metabolic pathway, synthesis of protein and protein refolding [27,28,29], which further confirmed the above hypothesis. Because of the presence of these cold resistant heterotrophic nitrifying bacteria, BAF could maintain strong nitrification ability at low temperature. 

As can be seen from Table 5 and Figure 5, DGGE bands 1, 2, 3, 4, 9 and 12 show 98%, 96%, 100%, 100%, 98% and 100% similarity to *Dechloromonas agitata*, *Myxobacterium AT3-03*, *Pseudomonas sp*, *Thauera aminoaromatica*, *Comamonas granuli* and *Rubrivivax gelatinosus* [30,31,32,33,34,35], respectively, and all of these bacterial strain exhibited denitrification ability. DGGE bands 1 and 4, on behalf of the denitrifying bacteria *Dechloromonas agitate* and *Thauer aaminoaromatica*, were only found in Lane 1, which indicates that these two kinds of microorganisms can survive only in relatively high temperature conditions. DGGE bands 2 and 3 on behalf of *Myxobacterium AT3-03* and *Pseudomonas sp.* can degrade the polymers into small molecules to provide the necessary organic carbon for denitrification [31,36]. With the decrease of temperature, the brightness of the strip 2 and 3 in each lane also decreased (Figure 3), indicating that the biomass of these two kinds of microorganisms declined with the decrease of temperature, but the microbial composition did not change, making it possible for the two kinds of microorganisms to degrade polymers to provide the necessary organic carbon for denitrification under different temperature conditions. Yao et al. [37] also found that *Pseudomonas sp.* played a major role in denitrification under low temperature. *Comamonas granuli* (band 8) was a common denitrifying bacterium in solid-phase denitrification process [38]. The brightness of strip 8 decreased with the temperature firstly and then disappeared from the lane, indicating that *Comamonas granuli* had poor ability on cold tolerance, and therefore can only survive in a certain temperature range. DGGE band 12, on behalf of *Rubrivivax gelatinosus,* represented the predominant strain, the biomass of which changed very little during the temperature changing process, suggesting that the microorganism has stronger resistance to low temperature. As can be seen from Figure 3, when the temperature decreased from 26 to 20 °C, no obvious change was observed in the influent nitrate concentration of SPDB, but the effluent nitrate concentration increased significantly. According to the change of microorganism biomass and microbial composition with the temperature mentioned above, the disappearance of some denitrifying bacteria (band 1 and 4) and biomass decreasing of polymers degrading denitrifying bacteria (band 2 and 3) might be the main course for the deterioration of denitrification. When the temperature continued to decrease from 20 to 13 °C, the nitrate concentration in the effluent of SPDB increased further, indicating that the denitrification performance continued to deteriorate. Although low temperature resistant aerobic denitrifying bacteria *Acinetobacter* sp. (band 11) and *Bacillus* sp. (band 13) [37,39] gradually developed into dominant bacteria with decreasing temperature, and the negative effect of denitrifying bacteria *Comamonas Granuli* (band 8) disappeared from the strip on denitrification was therefore compensated, the further biomass decrease of polymers degrading denitrifying bacteria *Myxobacterium AT3-03* significantly reduced the organic carbon release. Due to the insufficient electronic donors supply, denitrification in SPDB was greatly inhibited. 

### 3.4. Characterization of Biofilm Carriers under Different Temperature by ESEM

**Figure 6:**
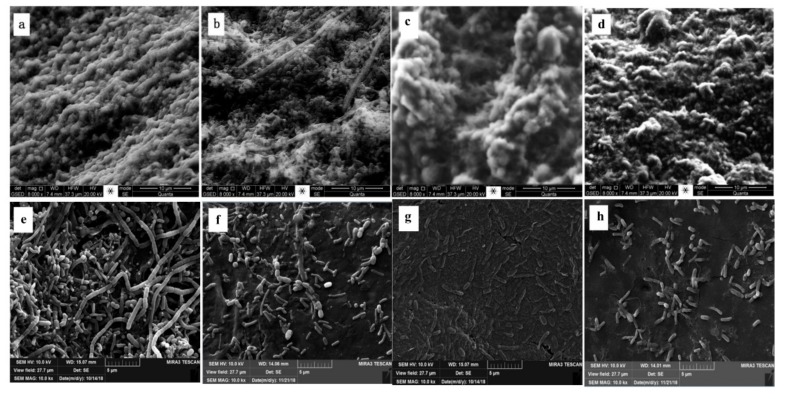
Environmental scanning electron microscope (ESEM) observations of surface of clay ceramsite with biofilm under (**a**) 26 °C, (**b**) 20 °C, (**c**) 18 °C, (**d**) 13°C and surface of PCL carrier with biofilm under (**e**) 26 °C, (**f**) 20 °C, (**g**) 18 °C, (**h**) 13 °C.

To track the biofilm development on the clay ceramsite and PCL carriers and elucidate the effect of temperature on biofilm structure, ESEM observations were conducted under the temperature of 26 °C, 20 °C, 18 °C and 13 °C. Figure 6 provides an impression of the surface structure and biofilm coverage on the outer surface of the clay ceramsite and PCL carriers during the temperature changing. As seen in Figure 6, with the decrease of temperature, the biomass attached to the surface of clay ceramsite and PCL carriers decreased gradually. The biofilm attached was thick and dense when the temperature was 26 °C, but when the temperature decreased from 26 °C to 13 °C, the biofilm became thinner and sparse, leading to the part-exposure of the bare carrier surface. Temperature had little effect on the morphology of microorganisms on the surface of clay ceramsite (Figure 6a–d), but had significant effect on that of the PCL carriers. From Figure 6e–g, it can be seen that spheroidal bacteria and rod-shaped bacteria are the main microorganisms on the surface of PCL carriers when the temperature was 26 °C, but the number of spheroidal bacteria decreased with the decreasing temperature. When the temperature was 18 °C, rod-shaped bacteria was the main microorganism on the surface of PCL carriers, and almost no spheroidal bacteria existed. This was consistent with the previous DGGE analysis results which showed that *Acinetobacter* sp. and *Bacillus* sp., two rod-shaded bacteria, gradually developed into dominant bacteria when the temperature decreased to 13 °C. 

## 4. Conclusions

- In the CS-BAF-SPDB combined process, to realize favorable nitrifying and denitrifying performance simultaneously in the BAF-SPDB unit, the temperature should be controlled above 18 °C when the influent C/N ratio and gas/water ratio of BAF is 3:1 and 4:1, respectively, and HRT of BAF and SPDB is 3.5 h and 1.5 h, respectively. The NH_4_^+^-N and TN removal efficiency of BAF-SPDB unit can reach more than 96.2% and 81.2% when the temperature is above 18 °C.
- In addition, the influence of the macro operation parameters on the performance of the BAF and SPDB has a direct relationship with the dynamic changes of the micro microbial community. The influence of temperature on nitrification performance in BAF is mainly embodied in the change of composition, amount and activity of ammonia oxidizing bacteria *Candidatus Nitrospira defluvii* and nitrite oxidizing bacteria *Nitrosomonas* sp. Denitrification performance of *Nm47* in SPDB is mainly embodied in the change of composition and amount of solid carbon substrate degrading denitrifying bacteria *Pseudomonas* sp., *Myxobacterium AT3-03* and heterotrophic denitrifying bacteria *Dechloromonas agitate*, *Thauera aminoaromatica*, *Comamonas granuli* and *Rubrivivax gelatinosus*. Meanwhile, the change of microbial community structure of heterotrophic nitrification and aerobic denitrification with operation conditions also plays a very important role in ensuring the stable nitrification and denitrification under low temperature.

## Figures and Tables

**Table 1:** Physical–chemical properties of the two biofilm carriers used in the experiment. Polycaprolactone, PCL.

Carrier Type	Product Mark	Appearance Shape	Density (g/mL)	Diameter (mm)	Height (mm)	Molecular Weight (Dalton)
Clay Ceramsite	PP-B 3.0	pellet	1.67	4–6	-	-
PCL	1400C	cylinder	1.08	3	4	140,000

**Table 2:** Universal Primer information.

Primer	Sequence
338F	CCT ACG GGA GGC AGC AG
518R	ATT ACC GCG GCT GCT GG
GC338F	CGCCCGGGGCGCGCCCCGGGGCGGGGCGGGGGCGCGGGGGG CCT ACG GGA GGC AGC AG

**Table 3:** Denaturing Gradient Gel Electrophoresis (DGGE) gel formulations.

Reagent	35%	55%
30% Acrylamide/Bis	4 mL	4 mL
50 x TAE buffer	0.3 mL	0.3 mL
Formamide (deionized)	2.1 mL	3.3 mL
Urea	2.205 g	3.465 g
dH2O	To 15 mL	To 15 mL
APS	120 μL	120 μL
TEMED	10 μL	10 μL

**Table 4:** The analysis results of DGGE gel bands recovery sequence in BAF.

The Analysis Results of DGGE Gel Bands Recovery Sequence				
Band Number	Similar Strain	Accession Number	Similarity	Classification
Band1	Labilithrixluteola	NR_126182	98	Proteobacteria Labilithrix
Band2	uncultured bacterium	KC797661	99	Bacteria; environmental samples
Band3	Dechloromonasagitata	KF800710	98	Proteobacteria Dechloromonas
Band4	Lactococcus sp. R.M17	HG937722	100	Firmicutes Lactococcus
Band5	Rubrivivaxgelatinosus	KF911343	99	Proteobacteria Rubrivivax
Band6	Gracilibacteria bacterium oral taxon 872	JX294353	98	Bacteria; Gracilibacteria
Band7	Bosea sp.	AB974256	100	Proteobacteria Bosea
Band8	Nitrosomonas sp. Nm47	AY123810	95	Proteobacteria Nitrosomonas
Band9	Acinetobacter sp.	KP636746	100	Proteobacteria Acinetobacter
Band10	Acinetobacter calcoaceticus	KR856228	100	Proteobacteria Acinetobacter
Band11	CandidatusNitrospiradefluvii	NR_074700	99	Nitrospirae Nitrospira
Band12	Acinetobacter bouvetii	KJ865593	100	Proteobacteria Acinetobacter
Band13	Bacillus sp.	KT452789	100	Firmicutes Bacillus

**Table 5:** The analysis results of DGGE gel bands recovery sequence in SPDB.

The Analysis Results of DGGE Gel Bands Recovery Sequence				
Band Number	Similar Strain	Accession Number	Similarity	Classification
Band1	Dechloromonasagitata	KF800710	98	Proteobacteria Dechloromonas
Band2	Myxobacterium AT3-03	AB246770	96	Proteobacteria Myxococcales
Band3	Pseudomonas sp.	KP711533	100	Proteobacteria Pseudomonas
Band4	Thaueraaminoaromatica	FJ609688	100	Proteobacteria Thauera
Band5	uncultured bacterium	FJ229147	96	Bacteria; environmental samples
Band6	Bosea sp.	AB974256	100	Proteobacteria Bosea
Band7	Nitrosomonas sp. Nm47	AY123810	95	Proteobacteria Nitrosomonas
Band8	Acinetobacter sp.	KP636746	100	Proteobacteria Acinetobacter
Band9	Comamonasgranuli	NR_114013	98	Proteobacteria Comamonas
Band10	Acinetobacter haemolyticus	KT260794	100	Proteobacteria Acinetobacter
Band11	Acinetobacter sp.	KT361093	100	Proteobacteria Acinetobacter
Band12	Rubrivivaxgelatinosus	NR_074794	99	Proteobacteria Rubrivivax
Band13	Bacillus sp.	KT452789	100	Firmicutes Bacillus
Band14	Thiobacillusaquaesulis	LN794608	99	Proteobacteria Thiobacillus
